# Towards neuro-inspired symbolic models of cognition: linking neural dynamics to behaviors through asynchronous communications

**DOI:** 10.1007/s11571-017-9435-3

**Published:** 2017-04-01

**Authors:** Pierre Bonzon

**Affiliations:** Department of Information Systems, Faculty of HEC, University of Lausanne, 1015 Lausanne, Switzerland

**Keywords:** Cognitive architecture, Cognitive development, Symbolic model, Learning processes, Neural dynamics, Asynchronous communications, Virtual machine

## Abstract

A computational architecture modeling the relation between perception and action is proposed. Basic brain processes representing synaptic plasticity are first abstracted through asynchronous communication protocols and implemented as virtual microcircuits. These are used in turn to build mesoscale circuits embodying parallel cognitive processes. Encoding these circuits into symbolic expressions gives finally rise to neuro-inspired programs that are compiled into pseudo-code to be interpreted by a virtual machine. Quantitative evaluation measures are given by the modification of synapse weights over time. This approach is illustrated by models of simple forms of behaviors exhibiting cognition up to the third level of animal awareness. As a potential benefit, symbolic models of emergent psychological mechanisms could lead to the discovery of the learning processes involved in the development of cognition. The executable specifications of an experimental platform allowing for the reproduction of simulated experiments are given in “Appendix”.

## Introduction

### Necessity of a multilevel approach to cognition

From a functional perspective, the brain can be seen as a kind of computing machine relating input and output in a significant manner defining behaviors. Yet no basic instruction set is known for the brain, nor is any kind of addressable repository of instructions and data, which together would allow for defining this relation in a formal way. This machine obviously does not work as traditional computers, whose design still follows the concepts introduced by von Neumann in the 1940’s i.e., it does not involve a stored program acting on stored data. Interestingly enough, the usual way to simulate a brain today still follows pioneering work dating back from about the same time i.e., that of McCulloch and Pitts (1943) defining finite-state automata that implement a threshold logic, Hodgkin and Huxley (1952) using differential equations to simulate the electrical processes surrounding neurons, and Rall (1964) taking into account the dendritic trees to define neuronal input–output relations. In these approaches, the brain is considered solely as a physical substrate. By analogy, this would amount to restricting the study of a computer to the description of its electronic circuits, or hardware, ignoring its software level that expresses algorithms under the form of programs. Similarly to the way algorithms running on a computer do represent computation, one may then ask: could symbolic programs intended to represent cognition be implemented on top of a simulated brain substrate?

In a similar perspective, the “*what*” and “*how*” of cognitive science can be described using the historical “tri-level” hypothesis (Marr 1982) that distinguishes *computational*, *algorithmic* and *implementation* levels. According to (Poggio 2012), the original work that led to this hypothesis included first a *behavioral* level that was eventually replaced by the computational one (as noted by this author, this replacement was indeed influential in the development of computational neuroscience as we have witnessed it since). This same author further argues that, in order to discover the representations used by the brain, one needs to understand “*how* an individual organism learns and evolves them from experience of the natural world”, and that “learning algorithms and their a priori assumptions are deeper and more useful than a description of the details of *what* is actually learned”. As a consequence, *evolution* and *learning* should be added to the list of levels in cognitive studies.

Analogous conclusions about the necessity of a behavioral learning dimension in cognition can be found in the insightful review of van der Velde and de Kamps (2015), who argue that cognitive processes are executed in connection structures that link sensory circuits (i.e., perception) with motor (i.e., action). What is needed, they add, is “a mechanism that shows how the information (*synchrony* of activation in this case) can be used by the brain”. An argument very much related to this can be found in (Forstmann and Wagenmakers 2015). According to these authors, top–down approaches via analytical and/or abstract mathematical tools such as Bayesian inference rules (see e.g., Ma and Pouget 2008), and for that matter we may add the bottom-up approaches of the classical theories based on artificial neural networks (Kohonen 1982; Hopfield 1982; Rumelhart and McClelland 1986; for an introduction see, Anderson 1995) as well as methods related to dynamical systems theory (see e.g., Wright and Bourke 2013; for an introduction, see Vernon 2014), are well suited for describing computations in Marr’s sense, but *“fail to identify algorithms and underlying circuits”.* What is then needed, they conclude, is a ‘‘*middle*-*out*’’ approach that can identify plausible structures linking biology and cognition.

### Roadmap towards a “middle-out” approach

Looking at the brain as a computing device linking neural dynamics to behaviors has led to the emergence of quite a few related research domains. Whereas *computational neuroscience* addresses low level neural mechanisms that give rise to higher level processes representing computations, *cognitive neuroscience* attempts to relate brain and behavior by linking latent cognitive processes to the neural mechanisms that generate them (Frank and Badre 2015). These two disciplines, when taken together, form the *computational cognitive neuroscience* (or *CCN*) paradigm (O’Reilly and Munakata 2000; Ashby and Helie 2011), in which artificial neural network models and methods serve *both* to specify and to concretize theories (Herd et al. 2013). A cognitive model however doesn’t have to represent its underlying neuronal processes itself, as the present approach to CCN does, but could rather adds an intermediate explanatory layer between the neuronal and behavioral level (Mulder et al. 2014; Frank 2015), using formal models to connect findings from neuroscience to the cognitive processes at hand (Forstmann and Wagenmakers 2015). The interface between these various layers could be described using computer science methods that allow for a delineation and implementation of successive levels of complexity.

Among the concepts that could be applied towards this goal, two are of particular relevance, namely *concurrent communicating systems*, on one hand, and *virtual machines,* on the other. The notion of a concurrent communicating system, which can be used to model the interaction of objects obeying various communication protocols, reflects a high level view of a network of interactive neurons. The concept of a virtual machine interpreting a compiled code that differs from a processor’s native code constitutes the key mechanism that allows for interfacing high level abstract objects i.e., software, with their low level physical support i.e., hardware. Following classical results of computer science, symbolic expressions that have been compiled and then interpreted by a virtual machine get their operational semantics from the transitions they induce on the state of this machine. In the context of a multi-level model of brain structures and processes, this means that low levels physiological details could be ignored, and grounded models of cognition be formulated by relating input and output (i.e., perception and behavior) at a *symbolic level*.

Yet, we still don’t know what a neural code for relating perception and behavior might be, and how to discover it. A possible way towards designing and/or guessing such a code is to explore the emergence of cognition in animals and then to try and reproduce it in computational terms, an idea somehow related to the ideas put forward by Badre et al. (2015) in their proposed birectional interaction between animal and human studies. In order to follow a smooth pattern of evolution leading to human behavior, models should be developed in progressive steps starting with the simplest of animal behaviors. Towards this end, experimental results from comparative zoology could be used to identify *invariant fundamental traits* of animal cognition (Pepperberg and Lynn 2000). In parallel, advances in the neuroscience (Gerstner and Kistler 2002) should allow to abstract functionalities of synaptic plasticity into neurally plausible *microcircuits*. These could be used in turn to build *mesoscale circuits* (Badre et al. 2015) corresponding to neural assemblies supporting the basic cognitive functions just identified. These circuits would then constitute the building blocks of perception (Perin et al. 2011).

This is the path that we have followed. A new simulation framework along the lines just sketched above is proposed: as computer applications can be first programmed, then compiled and finally interpreted by a virtual machine running as a native program, animal behaviors will be similarly encoded, compiled and then interpreted by virtual neurological microcircuits representing a brain’s innate processes. As a consequence, there will be no reference to any specific neural network model, but instead a *step*-*wise refinement* of successive virtual machines will eventually relate actual brain processes to overt behaviors. In contrast to the usual approach of creating neural models of interactive brain areas to by quantitatively fitting data (i.e., where latent estimated parameters are being correlated with neural measures), the goal here is to construct a generative model of how behaviors can be interfaced with neural dynamics in order to try and discover the learning processes involved in the emergence of cognition.

### Potential benefits

The potential advantages of such a symbolic computational framework can be described as follows: while the proposed formalism constitutes a way of expressing cognitive operations, and therefore remains a psychological description rather than a physiological one, it does it by providing a clear interface between the two domains. More specifically, and according to a notable attempt in this direction (Jilk et al. 2008), “the various levels of description will remain necessary to explain the full range of phenomena”. However, instead of considering the hierarchical arrangement of multiple *neuronal layers* such as the hierarchy of visual cortical layers V1 → V2 → V4 → IT → …, as neuroscientists usually do, this is to be understood in the sense of a hierarchy of model entities such as cell(or neuron) → cell assemblies → cognitive states → behavior → …. In particular, while the idea of a synchronous activation of brain processes (Singer 1993) is generally accepted when it comes to describe the functioning of the cortex, it is questionable whether the same hypothesis applies to the cognitive level (Eliasmith 2013), for instance to solve the binding problem (Feldman 2013) that arises when trying to link perception and behavior. Actually, a counterview has been recently advocated by Zeki (2015), which suggests that “there is no central neural clock in the (visual) brain that synchronizes the activity of different processing systems”, and that more likely “activity in each of the parallel processing-perceptual systems of the visual brain is reset independently, making of the brain a massively asynchronous organ”. Concretely then, the results of activities in the different processing-perceptual systems might not be bound by physiological interactions between cells in the specialized visual areas, but post-perceptually and asynchronously, outside the visual brain. In other words, if there is no doubt that at the physiological level e.g., in the cortex, the activity is widely synchronous, the description of the cognitive operations taking place at the psychological level, and more precisely their link with the underlying concrete neural circuitry, could be asynchronously driven. Again, as noted above, symbolic models of such mechanisms could lead to the discovery of the learning processes involved in the development of cognition. To support this hypothesis, our own work does rely on a bidirectional, or interactive, approach (see e.g., O’Reilly and Munakata 2000), where *bottom*-*up (*i.e., working from biological facts up to cognition) and *top*–*down* (i.e., working from cognition constraints down to biological facts) processes interact in coordination, in our case through *asynchronous* communications.

As a final word of introduction, let us stress here the exploratory nature of this work, which by no means represents a truthful modeling of the brain, and as such does not constitute a definite and mature alternative to some of the more ambitious projects currently underway (de Garis et al. 2010). The results that are reported here can be summarized as providing

- a simulation of a functional model of a brain as a symbolic virtual machine
- a graphical formalism whose repetitive patterns could be identified as its neural circuits
- an experimental platform that allows for reproducing these simulations.

The whole approach is illustrated in the “Results” with examples of simulated behaviors exhibiting cognition up to the third level of animal awareness (Pepperberg and Lynn 2000). More complex models including a simple form of meta-cognition (Fleming et al. 2012; Templer and Hampton 2012) as well as the learning of transitive relations via a form of analogical reasoning (Gentner and Forbus 2011) will be found in a companion paper.

## Materials and methods

Our overall methodology can be described in the following terms:

- *mesoscale circuits* (which correspond to basic cognitive processes produced by evolution) must be first induced from observed behaviors in comparative zoology
- these mesoscale circuits are then compiled into virtual code to be interpreted by a virtual machine running on top of *microcircuits* implementing synaptic plasticity (or more precisely, by a virtual machine executing virtual code designed to implement synaptic plasticity
- by definition, such a virtual machine constitutes an interface which allows for defining mesoscale circuits independently of the way the underlying layers i.e., the microcircuits, are actually implemented. Mesoscale circuits thus somehow correspond to cognitive software running on top of a biological substrate.

Circuits stand for *cell assemblies* (Hebb 1949; Palm 1982). These assemblies constitute a theoretical framework, which in some of its extensions (see e.g., Knoblauch et al. 2005; Wennekers and Palm 2009; Huyck and Passmore 2013) offers functional explanations of phenomena by linking them to physiological processes. Virtual machines, which in broad terms emulate the execution of a program in language *S* on a system having its own language *L,* similarly allow for interfacing two domains. To be more precise, the concept of a virtual machine that we use here (i.e., as it is usually understood in theoretical computer science, as opposed to a more general concept pertaining to the sharing of resources in operating systems) allows for interpreting virtual object code *L* compiled from source code *S*, as in the case of the Java virtual machine interpreting Java byte code obtained from the compilation of Java source code. On one side, symbolic expressions *s ∈ S* will represent virtual *circuits* that correspond to invariant fundamental traits of animal cognition. On the other side, logical *implications l ∈ L* compiled from these symbolic expressions will be used to deduce *virtual machine instructions* implementing neural dynamics. We follow a bidirectional approach and present in turn the *bottom up* design of virtual circuits followed by the *top down* construction of a virtual machine.

### Bottom up design of virtual circuits

Our bottom up design of virtual circuits follows from experimental results relating simple animal behavior to actual neuronal activity. As a general evolution principle, organisms tend to devise and use “tricks” for their survival. The ability to evaluate a threat by learning predictive relationships e.g., by associating a noise and the presence of a predator, is an example of such tricks realized by *classical conditioning*, as illustrated below with the defensive reflex of *aplysia* (Kandel and Tauc 1965). The ability to assess and to remember the consequences of one’s own actions is another example of an associative learning providing survival advantages. In this case, *operant conditioning* (Skinner 1950) associates an action and its result, which can be *positive* or *negative*. Toward this goal, the organism will first receive either an *excite* or an *inhibit* feedback stimulus, corresponding for instance to a reward or punishment, respectively; it will then associate this feedback with an appropriate action, let say *accept* or *reject* a perceived item.

#### A case of classical conditioning

**Fig. 1:**
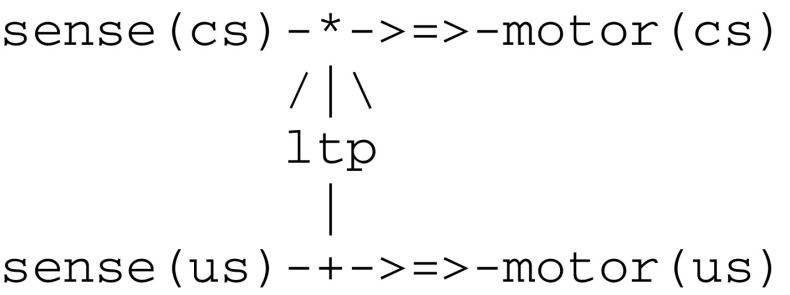
A virtual circuit implementing classical conditioning

Let us first consider an example of classical conditioning, where a light tactile conditioned stimulus cs elicits a weak defensive reflex and a strong noxious unconditioned stimulus us produces a massive withdrawal reflex. After a few pairings of stimuli cs and us, where cs slightly precedes us, a stimulus cs alone will trigger a significantly enhanced withdrawal reflex i.e., the organism has learned a new behavior. This can be represented by a wiring diagram, or *virtual circuit* (Fig. 1), adapted from Carew et al. (1981) to allow for a one to one correspondence with symbolic expressions.

In Fig. 1, the components sense(us) and sense(cs) are coupled with sensors (not shown here) capturing external stimuli us and cs and correspond to sensory neurons. The components motor(us) and motor(cs) are coupled with action effectors (also not shown) and correspond to motor neurons. Finally, the component ltp embodies the mechanism of long term potentiation and acts as a facilitatory interneuron reinforcing the pathway (i.e. augmenting its *weight*) between sense(cs) and motor(cs). The interaction of these components are represented by the iconic symbols ->=>- and/|\ that correspond to a synaptic transmission (i.e., ->=>- represents a *synapse*) and to the modulation of a synapse, respectively. The symbols * and + stand for conjunctive and disjunctive operators (i.e., they are used to represent the convergence of incoming signals and the dissemination of an outgoing signal, respectively). Classical conditioning then follows from the application of a hebbian learning principle i.e., “neurons that fire together wire together” (Hebb 1949; Gerstner and Kistler 2002).

Though it is admitted today that classical conditioning in aplysia is mediated by multiple neuronal mechanisms (Glanzman 1995; Antonov et al. 2003) including a postsynaptic retroaction on a presynaptic site, the important issue is that the learning of a new behavior requires a conjoint activity of multiple neurons. This activity in turn depends critically on the temporal pairing of the conditioned and unconditioned stimuli cs and us, which in conclusion leads to implement the ltp component as a *detector of coincidence*.

#### A simple case of operant conditioning

**Fig. 2:**
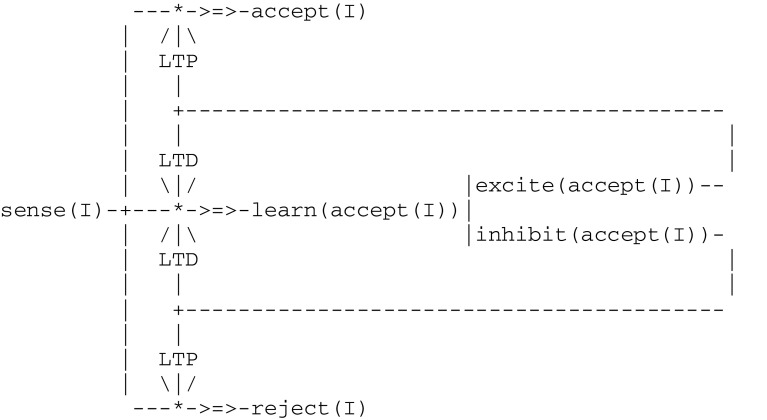
A generic virtual circuit implementing simple operant conditioning

Let us now consider a simple thought experiment where a *pigeon* is probing food, e.g., is learning to discriminate between items such grains and pebbles. Let us assume that for each item he perceives, his external visual stimuli consist of a vector I = [I
_1_
,I
_2_
,.] of primitive *features* (e.g., vectors [mat,smooth] and [shiny,smooth] could correspond to grains and pebbles, respectively). The *generic circuit* given in Fig. 2, where I stands as a parameter, represents the wiring of four components sense(I), learn(accept(I)), accept(I) and reject(I), together with two ltp and two opposite ltd (for long term depression) components. In addition to the external stimuli captured by component sense(I), this circuit incorporates the two internal stimuli excite(accept(I)) and inhibit(accept(I)) that correspond to feedbacks from probing the food according to a set of *accept* elements.

This generic circuit will give rise to an instantiated circuit for each possible vector I. At the beginning of the simulation, and for any I, the pathway from sense(I) to learn(accept(I)) is open, while the pathways to both accept(I) and reject(I) are closed. After a few trials, the pigeon will no longer probe his food, i.e., he will have learned to close the pathway to learn(accept(I)) and to open either accept(I) or reject(I), associating thus each input vector I with an action. With regard to the hypothetical neurological substrate corresponding to this scheme, let us just mention that this process matches some recent results from neuroscience, where emergent pictures of the brain are based on the existence of

- two eligibility traces with different temporal profiles: one corresponding to the induction of ltp, and the other to the induction of ltd (Huertas et al. 2014; He et al. 2015)
- two populations of neurons that have opposing spiking patterns in anticipation of movement suggesting that these reflect neural ensembles engaged in a competition (Zagha et al. 2015)
- a fundamental principle in circuit neuroscience according to which inhibition in neuronal networks during baseline conditions allows in turn for disinhibition, which then stands as a key mechanism for circuit plasticity, learning, and memory retrieval (Letzkus et al. 2015).

As a remark that will apply to all models introduced below (thus illustrating our methodology), each of the components contained in this generic model does represent neural assemblies whose detailed structures should be in turn modeled by a *step*-*wise refinement* of successive virtual machines eventually relating actual brain processes to overt behaviors.

#### Representing circuits by symbolic expressions

The basic entities of the proposed formalism for representing circuits are constituted by *threads*. In Computer science, a thread is a sequence of instructions that executes concurrently with other threads, may coexist with other threads to form a process and share resources such as memory. In the present context, a thread corresponds to a single or a group of neurons and will be represented by a symbolic expression enclosing an *instruction tree* (see below for the definition of the corresponding formal language *S*). Threads are communicating entities. Each communication does involve a pair of threads and entails on one side the signal transmitted by a pre-synaptic *(source*) thread, and on the other side its reception, via a given synapse, by a post-synaptic (*recipient*) thread. Similarly to a neuron, a thread can be both a source and a recipient and functions as a gate receiving incoming signals from different sources and sending an outgoing signal to possibly many recipients. There are however two essential differences between threads and neurons that allow for a single thread to represent a group of neurons i.e.,

- contrary to a neuron that alternates roles in cycles, a thread can be simultaneously a source and a recipient by maintaining parallel communications.
- contrary to traditional neuron models in which incoming signals are summed in some way into an integrated value, thread inputs can be processed individually.

**Fig. 3:**
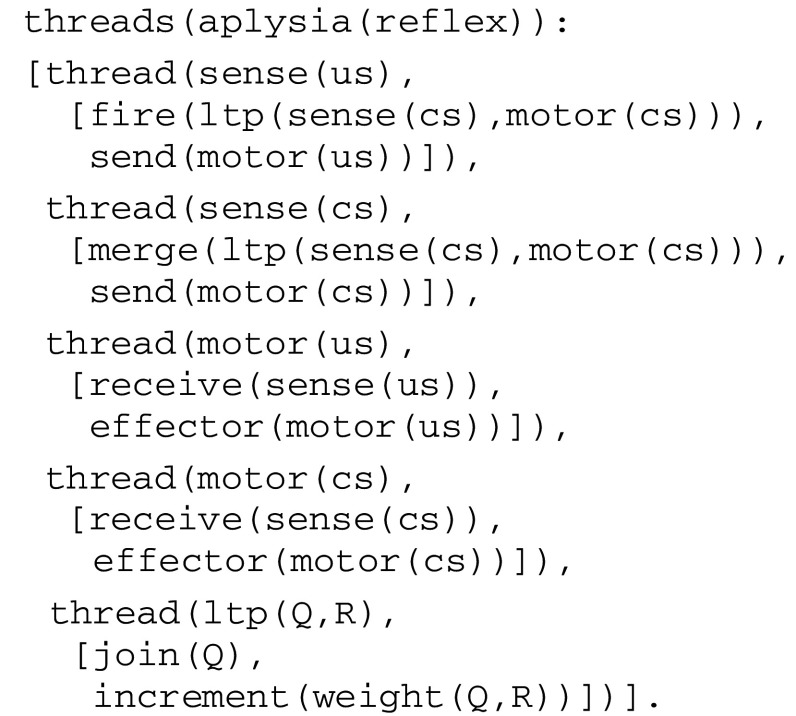
Fiber expression corresponding to the virtual circuit for classical conditioning

Threads can be grouped into disjoint sets, or *fibers,* to model neural assemblies, and discrete *weights* (e.g., integer numbers) can be attached to pairs of communicating threads belonging to the same fiber. In some sense, fibers correspond to the formal notion of independent *processes* made of concurrent threads. The interaction of threads obeys various communication *protocols.* These protocols will be implemented by means of procedures that operate in pairs. As an example, the protocol depicted by the symbol ->=>- corresponding to a synaptic transmission is implemented by a send/receive pair, and the symbol/|\ corresponding to the modulation of a synapse is implemented by a join/merge pair. A thread named Thread will be represented by a symbolic expression having the format thread(Thread,
*Tree*
), where *Tree* is an *instruction tree*. Similarly, a named fiber in a named model will be represented by an expression threads(Model(Fiber):
*List*
), where *List* is a list of thread expressions. As an example, the circuit in Fig. 1 gives rise to the fiber expression given in Fig. 3.

**Fig. 4:**
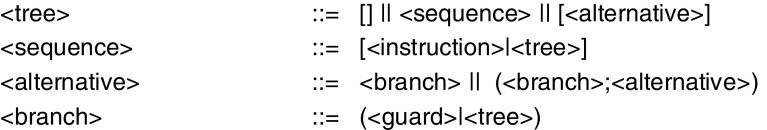
Production rules for instruction trees

In this simple example, the instruction tree associated with each thread reduces to a *sequence* (or linear list) of virtual instructions such as fire, send, merge, etc. As another example illustrated in Fig. 2, an instruction tree can contain an *alternative* (e.g., as in the thread try that has two branches commanded by a guard). Formally, symbolic expressions representing instruction trees belong to a language *S* whose syntax is defined by the production rules given in Fig. 4.

Whereas the non-terminal symbol <guard> represents conditions derived from internal stimuli (e.g., as a result of neurotransmitters), <instruction> stands for virtual machine *instructions* such as fire, send, merge, etc. (see the “Appendix” for a definition of this instruction set). This language *S* of instruction trees is not to be confused with the language *L* that will be used to define *virtual code implications* (and more generally the state of a virtual machine, see “Top down construction of a virtual machine” section) into which instruction trees will be then compiled, as illustrated below.

#### Compiling instruction trees into virtual code implications

Virtual code implications are compiled from thread expressions and have the following

- *Guard* => *T:Instruction*

where *Instruction* is a virtual machine instruction and *T* its clock time. As an example, let us consider the thread sense(us) in Fig. 3:
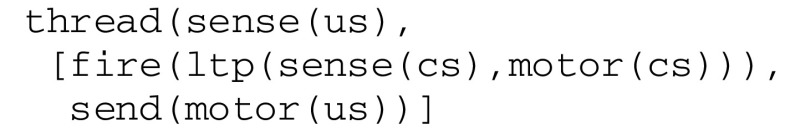


The straightforward virtual code implications compiled from this thread are:
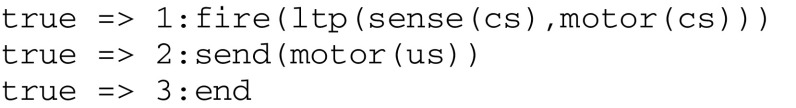


In this simple example, successive clock time values (i.e., 1, 2, 3) correspond to a linear list traversal. As another example, the thread learn(accept(I)) from Fig. 2, whose instruction tree contains an alternative giving rise to the following expression 
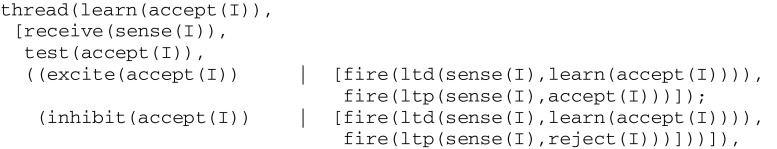
will be compiled into the following virtual code implications, whose repetitive successive clock values correspond to possible descends into a tree: 
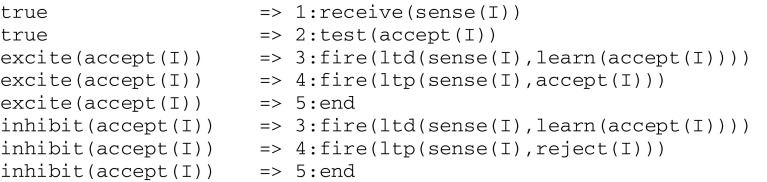


Generally speaking, the compilation of virtual code implications can follow from a recursive descent into an instruction tree (see “Formal specifications” sections).

### Top down construction of a virtual machine

Let us consider a set of *fibers* together with sets of initial *weights* for pairs of communicating threads within fibers and sets of *accept* elements in fibers. A fiber containing at least one active thread i.e., a thread whose associated clock is up and running, constitutes a *stream*. The virtual machine consists of

- a set of *registers* comprising, for each active thread, a local *clock* and four internal *stimuli* registers (i.e., *fetch, catch, excite, inhibit*) holding one value at a time
- a set of local *signal* queues attached to active threads and holding multiple values at a time
- a *content addressable memory* holding the virtual code implications attached to threads, as well as the sets of current weights and accept elements.

Let *Model* designate the state of the virtual machine as described in a language *L*. At the top level, the virtual machine is defined by a *run* procedure that consists of a *loop* whose cycle comprises a *sense* procedure followed by a *react* procedure:
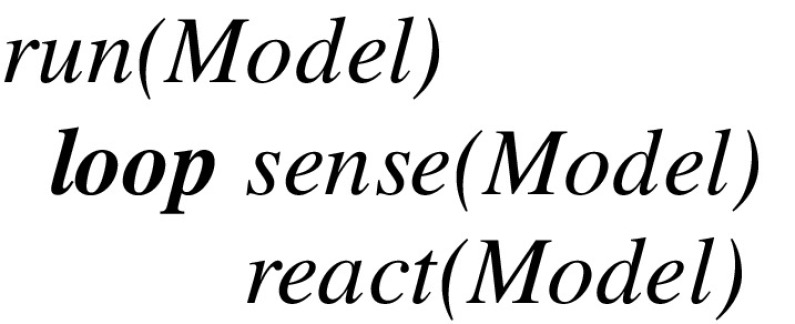


At the next level below, the *sense* procedure reflects the triggering of spike trains directed to sensory neurons. After possibly capturing an interrupt from sensors directed to a given stream (which initially can be a fiber i.e., without any active thread), it updates *Model* using a transition function *input*:
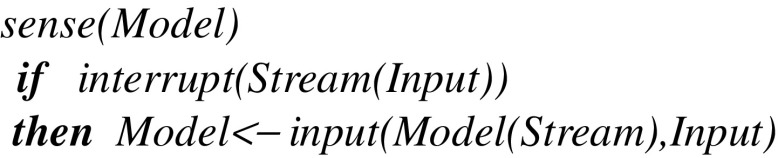


The transition function *input* first terminates the interrupted stream by clearing all its registers and queues and then resets the clocks of the sensory threads associated with sensors.

The *react* procedure itself consists of a loop calling on each active thread in any stream to first deduce a virtual machine instruction and then update *Model* using a transition function *output*: 
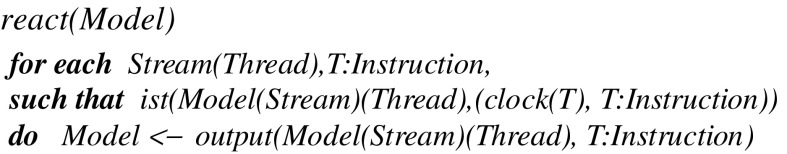


The transition function *output* corresponds to the execution of a virtual machine instruction and implements communication protocols to be specified in the “Microcircuits implementing synaptic plasticity” section. *T:Instruction* is deduced through *contextual deduction* (Bonzon et al. 2000) from virtual code implications that have been compiled from thread expressions and loaded into memory (see in “Computational architecture formal specifications” section for formal definitions, including that of the *ist* predicate standing for “is true”).

Clock register values *T*, which correspond to program counters in traditional virtual machines like the Java machine, are used to deduce, for each active thread, the next instruction satisfying the guard. Whenever a transition initiated by a thread succeeds, the thread clock is advanced and the next instruction is deduced and executed, and whenever it fails, the current instruction is executed again i.e., the transition is attempted until it eventually succeeds. Altogether, this amounts to descending into an instruction tree, with its local clock time corresponding to the currently reached depth, as illustrated in the previous section.

The mechanisms enforced in this virtual machine provide a solution to the problem of dynamically binding roles to filler (Hummel and Holyoak 2005). More precisely, this is achieved via both its sense procedure and the communication protocols between threads, which together amount to implementing a systematic asynchrony of firing as described in (Doumas et al. 2008). This stands in contrast with the usual approach to binding achieved through synchronized firing across separate but interconnected areas of the brain (Treisman 1996; Feldman 2013).

Before proceeding to a detailed specification of this machine, let us briefly summarize its salient features and their relation to a possible macroscopic view of the brain:

- contrary to traditional stored-program computers, this machine doesn’t have an instruction register holding the current instruction being executed after its retrieval from an addressable memory; by interpreting code deduced *just in time* from virtual implications compiled themselves from thread configurations that are akin to brain states, the overall architecture of this system could turn out to be closer to that of a brain.
- virtual code implications are reminiscent of *daemons* that run as computer background processes and are triggered by foreground application software; daemons were in common use in the early days of the Artificial Intelligence paradigm, when Neuroscience didn’t yet provide a neural substrate for models of perception and cognition (Powers 2015).
- similarly to machine code compiled from application software, this new kind of daemons is compiled from thread fibers that are thus akin to cognition software
- finally, as described above, these daemons are triggered by *local* deductions within a given stream; *global* deductions at the model level (to be introduced below) will give access, from within any stream, to previously active threads that will thus achieve the status of a global memory (see “Microcircuits implementing synaptic plasticity” sections).

## Results

We first report on the *neural* aspects of this work by presenting microcircuits implementing synaptic plasticity. We then detail a *computational* architecture by presenting the formal specifications of a virtual machine under the form of enhanced Prolog code. Finally, we include examples of mesoscale circuit modeling the first three levels of animal awareness.

### Microcircuits implementing synaptic plasticity

As illustrated in “Materials and methods” section, circuits rely on communication protocols that are pictured in thread diagrams by iconic symbols representing themselves microcircuits. These protocols can be defined by means of procedures that operate in pairs:

- send/receive denoted by the symbols
->=>- or -<=<-

represent synaptic transmission

- join/merge denoted by
  - /|\ or \|/

implement long term potentiation/depression (ltp/ltd)

- push/pull denoted by
-<A>-

model a short term cache memory (stm)

- store/retrieve denoted by
–{P}-

model an associative memory (ltm) based on long term storage and retrieval (lts/ltr).


These protocols are detailed below together with the corresponding microcircuits. The definition of the basic threads implementing these microcircuits is given in the “Appendix”.

#### Synaptic transmission

The microcircuit implementing a synaptic transmission i.e.,
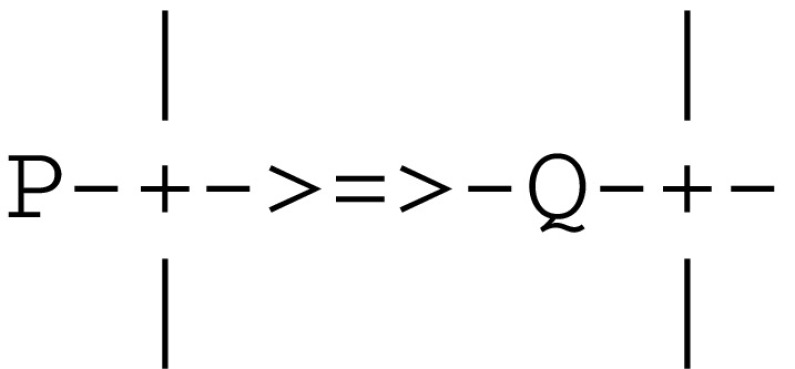


can be represented by the following expressions
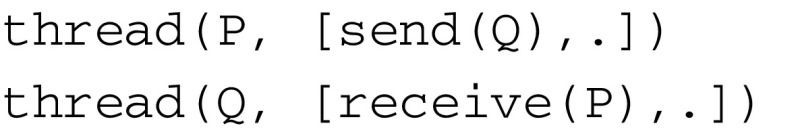


**Fig. 5:**
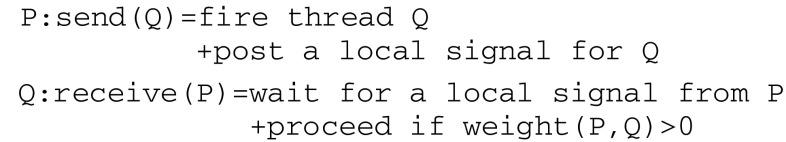
Communication protocol for synaptic transmission

The calls send(Q) and receive(P) correspond to the transmission of a local signal by a pre-synaptic neuron P followed by its reception by a post-synaptic neuron Q and are used to model local communications within a given stream. The firing of P is assumed to have occurred earlier e.g., in reaction to the capture of an external stimulus. These expressions give rise to the *communication protocol* given in Fig. 5.

This send/receive protocol corresponds to an *asynchronous* communication subject to a threshold. It involves a predefined weight between the sender P and the receiver Q. This weight can be incremented/decremented by an ltp/ltd thread. After firing thread Q and sending it a signal, thread P goes on executing its next instruction. On the other side, thread Q waits for the reception of a signal from thread P and proceeds only if the weight between P and Q stands above a given threshold. In any case no data is passed between the two threads, and the overall process just amounts to allowing Q to proceed on behalf (or at the demand) of P.

#### Long term potentiation/depression (ltp/ltd)

**Fig. 6:**
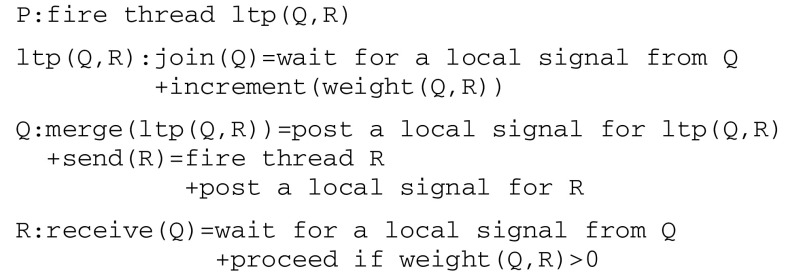
Communication protocol implementing long term potentiation

The join/merge pair is used in conjunction with the send/receive pair in order to implement the modulation of a synapse. The following microcircuit
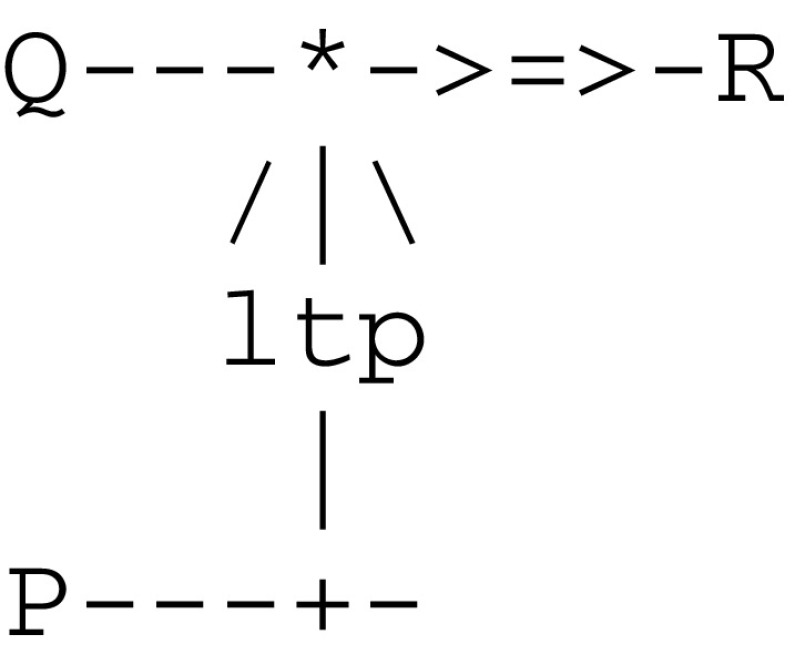
implementing *ltp* gives rise to the protocol given in Fig. 6.

An *ltd* thread can be similarly implemented by decrementing weights. As an example, and according to the experimental protocol of classical conditioning (cf. Fig. 1), one must first detect the pairing of the two stimuli cs and us. Towards this end, sense(us) fires an ltp thread that in turn calls on a join thread to wait for a signal from sense(cs). In parallel, sense(cs) calls on a merge thread to post a signal for ltp and then executes a send(motor(cs)) command to motor(cs). When met by sense(cs), thread ltp eventually increments the weight between sense(cs) and motor(cs).

#### Short term cache memory (stm)

**Fig. 7:**
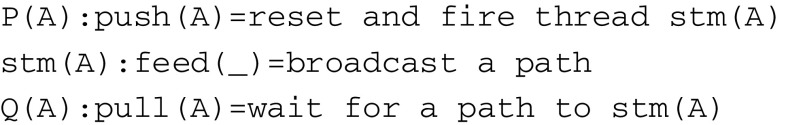
Communication protocol implementing a short term cache memory

As introduced in a model for the second level of animal awareness (Pepperberg and Lynn 2000), cache memory allows for remembering a location A. This can be represented by
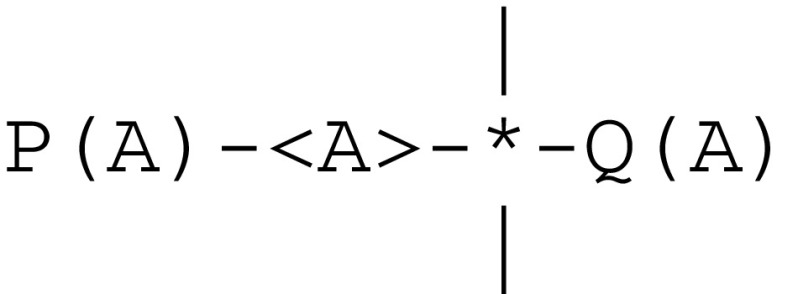
which gives rise to the protocol in Fig. 7.

Resetting stm(A) means that the previous value of A is no longer available. Furthermore, *broadcasting* a path, which amounts to posting a global signal, means that it can be received by any thread Q attached to any stream.

#### Associative long term memory (ltm) based on long term storage and retrieval (lts/ltr)

**Fig. 8:**
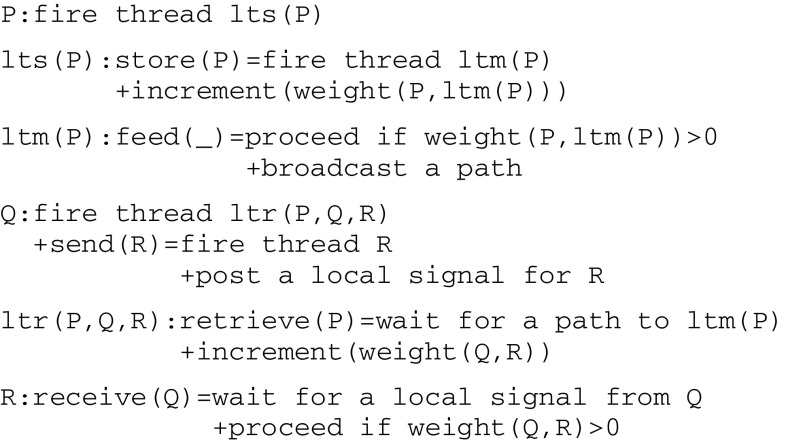
Communication protocol implementing a long term associative memory

According to Pepperberg and Lynn (2000), an organism having the third level of awareness must be able to *recall* properties of actual objects from previous perceptions. This implies in turn some kind of associative long term memory. The concept of an associative memory has been studied from various perspectives (see e.g., Palm 1980). In this particular context, an associative memory extends the mechanism of long term potentiation by allowing for two threads P and Q attached to separate streams (and thus also possibly active at different times) to be associated in order to trigger a *recall* thread R. These two streams will be linked together via a long term memory ltm(P)thread embedded in a microcircuit driven by a double communication protocol depicted by -{P}-.This new protocol involves two complementary *long term storage/retrieval* (lts/ltr) threads that allow for the building of a storage trace and a later retrieval of previously active threads. This is well in line with results by Rubin and Fusi (2007) demonstrating that if the initial memory trace in neurons is below a certain threshold, then it cannot be retrieved immediately after the occurrence of the experience that created the memory. This can be represented by the following microcircuit:
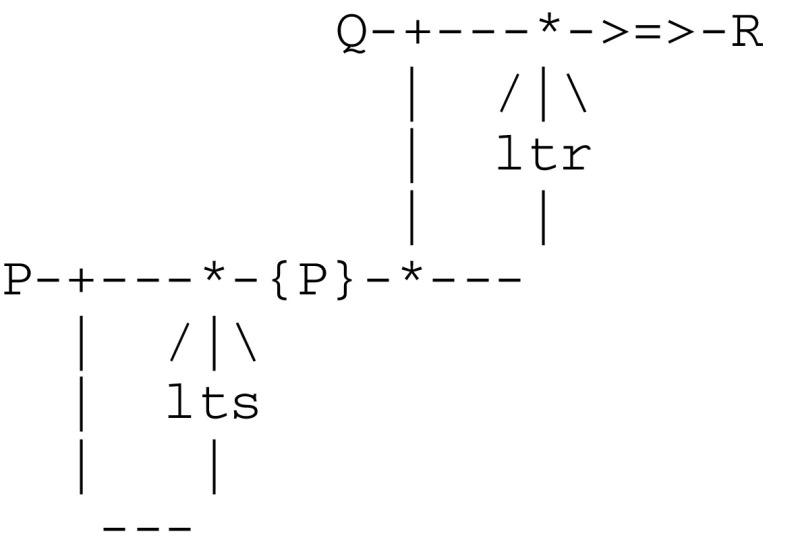
This microcircuit gives rise to the communication protocol in Fig. 8.

As a distinctive difference from an ltp(Q,R) thread (which gets fired by P and waits for a local signal from Q in order to relate Q and R), an ltr(P,Q,R) thread is fired by Q and waits for a path to ltm(P) in order to relate Q and R.


### Computational architecture formal specifications

The experimental platform allowing for running a virtual machine is now described in a top down approach.

#### Functional signatures

Virtual machines essentially emulate the execution of a program in language *S* on a system having its own language *L*. We recall the language *S* of instruction trees described in “Representing circuits by symbolic expressions” section. As for the language *L,* it allows for defining the state of the virtual machine itself (which we recall consists of sets of registers and queues together with a content addressable memory holding compiled virtual code implications as well as current weights and accept elements).

Compiling an instruction tree into a set of virtual code implications can be represented by a *compile* function with the following signature:

*compile: S → L*

Compiling and then loading a set of virtual code implications into a virtual machine leads to define a combined *load* function (actually the composition of the compile function with an insert function):

*load: S*.*× (S → L) × L → L*

This function can be easily extended to include loading the sets of initial weights and accept elements using a compile function equal to the identity function. Finally, let *I* and *O* be the languages defining *input/output* sentences captured by *sensors* and delivered to *effectors*, respectively. Running a model on a virtual machine then defines a *run* function as follows:

*run: I × S× (S → L) × L → L× O*

#### Formal specifications

A complete specification of this computational architecture is given below under the form of Prolog code, which at the same time provides an effective, even if not really efficient, implementation.

Language conventions used throughout include:

- identifiers starting with capital letters represent variables
- expression F(|X) represents a term with an arbitrary atomic functor F and any number of arguments e.g., F(|X) can be unified with p(1), f(a,b), etc.
- the character “ – ” represents a blank variable whose instantiation is not required.

This code is enhanced with macro definitions in order to improve its readability. Some of these language extensions (e.g., loop, interrupt, if then else, etc.) have an intuitive meaning and won’t be developed here. The others do represent an implementation of the formal notions of a *context* and of *contextual deduction*.

##### Implementing a context as a dynamic set of elements

We start with the implementation of a context defined as a dynamic set of elements associated with the following operations, where each *instance* plays the role of a non-logical axiom in a logical theory:
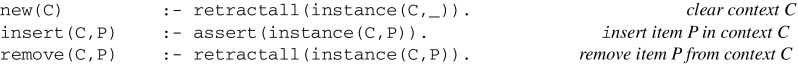
These operations also constitute our implementation of a *content addressable memory* as well as that of a *queue*, both being considered here as data buffers whose values can be accessed in any order.

##### Setting the value of a register in context

On this basis, a register holding one value at a time can be implemented by a single set operation:
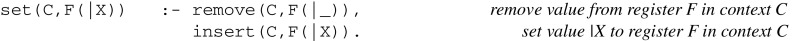


##### Contextual deduction

Given a context, the ist predicate standing for “is true in this context” is defined as follows:
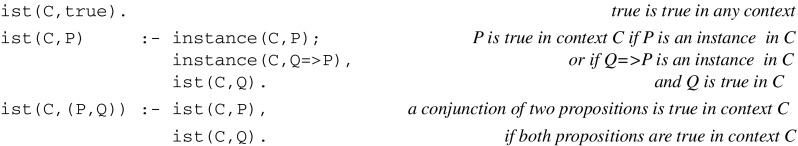


##### Compiling virtual code implications

The compilation of virtual code implications follows from a recursive descent into an instruction tree. According to its signature, the corresponding function can be implemented as a *compile* procedure with one input argument and one output argument standing for an instruction tree and compiled code, respectively:
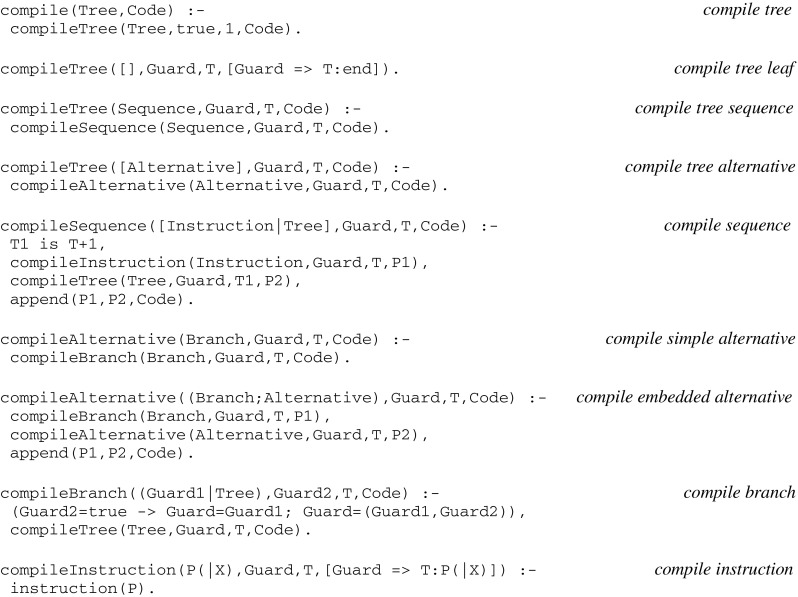


##### Loading a model

Instead of defining a *load* procedure whose arguments reflect the functional signature given above, let us extend the definition of a model (as introduced in “Top down construction of a virtual machine”, where *Model* designates a *state ϵ L*) in order to come up with a single argument, with *Model* designating now a *state ϵ S* × *L*. Let us then consider a set of fiber assertions as introduced in “Representing circuits by symbolic expressions” section i.e., expressions of the form

*threads(Model(Fiber)):[thread(Thread*_*1*_ *,Tree*_*1*_ *),..thread(Thread*_*n*_ *,Tree*_*n*_ *)]*.

together with global assertions for *basic* threads implementing synaptic plasticity and memory (see their definition at the end of the “Appendix”), as well as assertions for *initial* weights and *accept* elements. A combined load can be defined as follows:
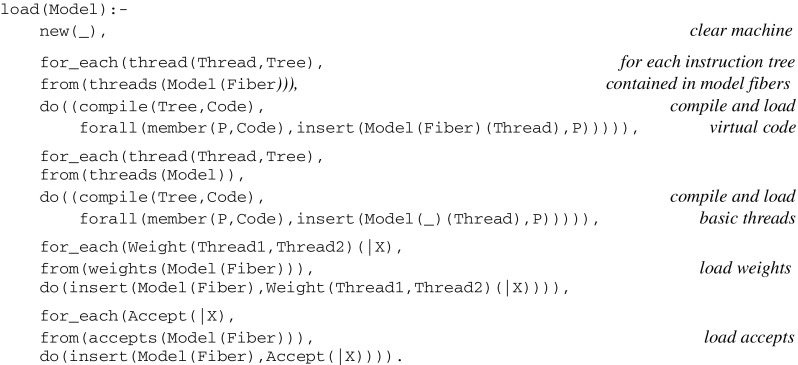


##### Running a model

Let *Interrupt,* the input sentence captured by *sensors* at successive run cycles, be represented by a list of the form

*[sensor(|X*_*1*_ *),..sensor(|X*_*n*_ *)]* .

The run function is then defined as follows:
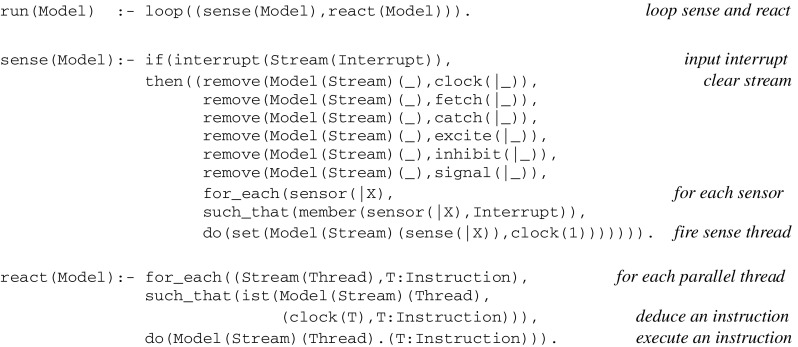


The formal specification of the set of virtual machine instructions is given in the “Appendix”.

### Examples of mesoscale circuits

We present operational models of simple animal behaviors that were executed on the experimental platform described in the previous section. These models offer simulations of the first three level of animal awareness according to Pepperberg and Lynn (2000). More complex models showing how a simple form of meta-cognition, namely memory awareness (Fleming et al. 2012; Templer and Hampton 2012), can be reduced to successive layers of associative memories implementing retrospective revaluation, on one hand, and another model implementing the learning of transitive relations via analogical inferences (Hummel and Holyoak 2005), on the other, will be found in a companion paper.

#### A model of the first level of animal awareness

Let us consider an example of operant conditioning that involves a choice between two alternatives. In an experiment (see e.g., Zentall et al. 1981), pigeons are first confronted with a lit sample that can be either red or green, and then must peck one of two lit buttons (say, one left and one right button). The color of the sample and of each button varies randomly from one trial to the next, but there is always one green button and one red button. In order to get a reward, a pigeon must peck the button that does have (or doesn’t have, according to the type of the experiment) the same color as the sample.

**Fig. 9:**
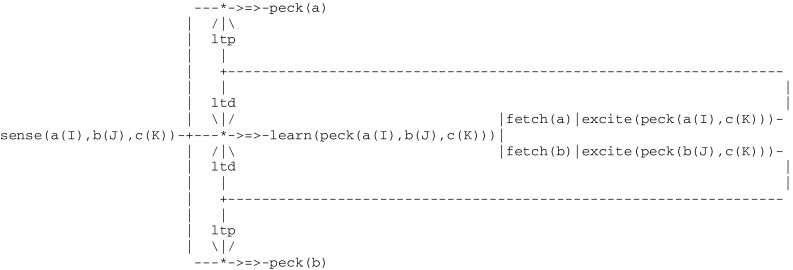
Circuit implementing operant conditioning with a choice

According to Wright (2001), there are two different ways that pigeons can learn matching to sample. Their first strategy is to associate each configuration (e.g., each combination of external stimuli) with the corresponding correct choice. This can be implemented by the circuit given in Fig. 9 that looks like a simple extension of the Fig. 2 implementing simple operant conditioning, where *positive* feedbacks only are taken into account (in order to simplify the presentation, the preliminary step involving the presentation of the sample alone is omitted):

Visual stimuli captured by sensors are represented by three expressions a(I), b(J), c(K), where a, b, c correspond to the left button, the right button and the sample, respectively, and the parameters I, J, K take the values green or red. In addition to these external stimuli, two internal stimuli i.e., fetch(a),excite(peck(a(I),c(K))) and fetch(b),excite(peck(b(J),c(K))) first command the choice made by the pigeon (i.e., either “left” or “right”, resulting from a random selection) and then provide a positive feedback when the choice was correct (i.e., the pigeon got rewarded). As an example, if the input configuration is a([green]),b([red]),c([green]) then the correct choice is fetch(a) leading to peck(a).

**Fig. 10:**
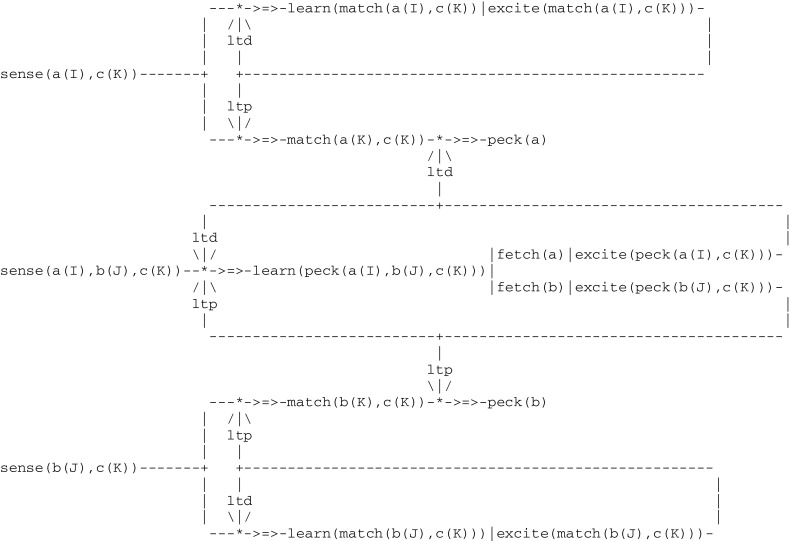
Circuit implementing the first level of animal consciousness

Another pigeon strategy to learn this task is as follows: while first randomly pecking either one of the two buttons, it does compare them in turn with the sample and learn to match colors. After a training period, it then stops pecking randomly and selects the button that does match the sample. In other words, pigeons do not learn to choose a color in a given arrangement of colors, but to match and then choose the match. According to Pepperberg and Lynn (2000), the first level of animal awareness, corresponds to the ability t*o follow a simple rule* involving the perception of a specific item or event and either its acceptation or its rejection. The second strategy that was just described, which can be characterized as learning *matching*/*oddity to sample,* does actually constitute an instance of the first level of awareness. This can be represented by a three layer circuit (Fig. 10). The middle layer implements the random pecking of a button. The two outer layers implement learning to match a button with the sample and eventually select the match. This overall circuit functions as an learning automaton that is being trained to accept one of two objects a and b whose color I and J does match the color K of the sample c. Note that the pathways to peck(a) and peck(b) are opened by an ltp thread initiated by the middle layer whenever a trial ends with a reward.

#### A model of the second level of animal awareness

In a nut shell, whereas the first level of animal consciousness does not allow for an immediate transfer to a similar task, an organism with the second level is aware enough of a rule to transfer it across situations (Pepperberg and Lynn 2000). In an experiment reported by (Cole et al. 1982), hummingbirds face the choice of visiting one of two locations potentially containing food. In order to solve this dilemma, they are trained to adopt a strategy that effectively relies on remembering the location they visited last. Along the same lines, it is known that rats do use specialized neuronal cells (which include *head direction, place* and *grid cells*) to create internal cognitive maps of their environment, direct themselves and remember places they have been visiting (see e.g., O’Keefe and Nadel 1978; Moser et al. 2008). Moreover, recent research indicates that these capabilities are innate (Langston et al. 2010; Wills et al. 2010). Let us extrapolate these results to hummingbirds. Each trial in this experiment consists of two separate stages. In the first (information) stage, an artificial flower containing food is presented in one of two possible locations i.e., in a cage left or right corner. After the bird is fed, the flower is removed. In the second (choice) stage, a flower is presented in each corner, one flower containing food and the other one empty. Birds are then allowed to visit one flower only. If the flower containing food consistently stands in the same corner as in the information stage, birds are thus required to return to the location they have just visited with success, which corresponds to adopt a *win/stay lose/shift* rule (or strategy).

**Fig. 11:**
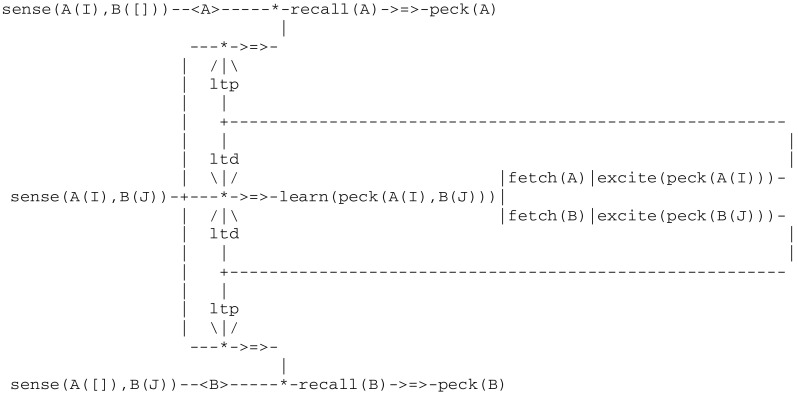
Circuit implementing the second level of animal consciousness

This experiment gives rise to the following model in Fig. 11.

This model has the following characteristics:

- an information stage can be initiated by one of two threads sense(A(I),B([])) and sense(A([]),B(J)), where the parameters A, B denote for example the left and right corner, the parameters I and J are the expression flower(food)signaling a flower with food, and “[]” signals a location without a flower
- the choice stage is initiated by a thread sense(A(I),B(J)), where I and J can be either one of two expressions flower(food)and flower([])corresponding to the location of a flower with food and without food, respectively
- these two stages are interconnected via a new interaction protocol denoted by -<A>- or -<B>- allowing for the short term cache memory (or stm) of location A or B.

When discussing this experiment, (Pepperberg and Lynn 2000) first note that an organism having a second level of awareness “is aware enough of the rule to transfer it across situations” (e.g., across inflorescences). This is reflected in the above model by parameters A and B allowing for the representation of various environments. They then add: “If, however, the organism were truly aware of using the rule, it would, when transferred to a *win/shift lose/stay* paradigm, readjust after only a few trials”, which actually they do not. This is reflected in the model by the fact that implementing the converse *win/shift lose/stay* strategy requires to consider negative inhibit feedbacks instead of the positive excite used above.

#### A model of the third level of animal awareness

The third level of animal awareness provides an organism with the additional capacity *to integrate two different sets of stored information*. In order for example to make a categorical judgment (e.g., to sort items), an organism has to *recall* properties of actual objects. This implies in turn some kind of *associative long term memory*. This can be illustrated through an experiment reported in (Savage-Rumbaugh et al. 1980). In the first phase of this experiment, chimpanzees were familiarized with a set of objects (such as a cake and an orange, on one hand, and a key and a stick, on the other) belonging to two categories i.e., *edible* and *inedible*. In a second phase, they were trained to sort a *subset* of objects of each kind by placing them in two different bins. The question that did then arise was to determine in which of two possible ways they learned this task i.e., by memorizing an association between each item and the appropriate bin, or by devising the rule “this bin is for items that I eat and the other bin is for items that I do not eat”. If such a rule had been be used, then the chimpanzees could sort more familiar objects of each kind without additional training. A subsequent test showed that this was indeed the case.

**Fig. 12:**
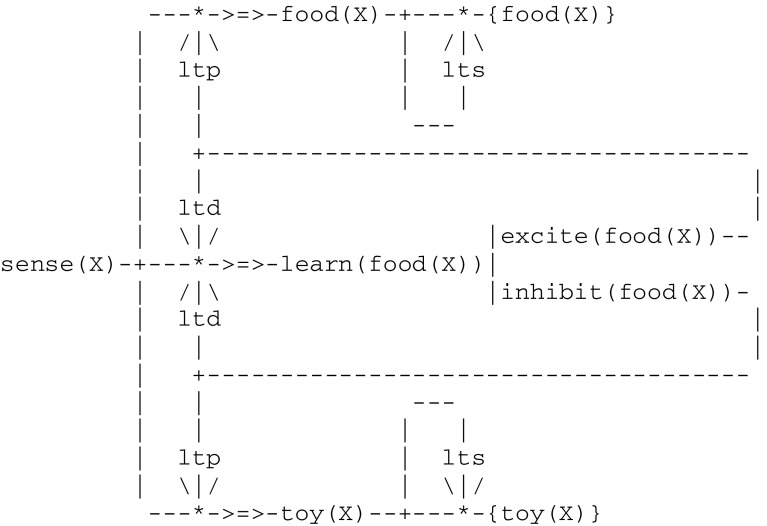
Circuit for memorizing a category

The two phases of this experiment can be implemented by two independent circuits, possibly active at different times. These two circuits are linked together by a double communication protocol depicted by -{P}- implementing an associative long term memory (or ltm). This protocol involves two complementary *long term storage/retrieval* (lts/ltr) processes that allow for the building of a thread storage trace and a later retrieval from this trace. The first phase (Fig. 12), which starts with the categorization of each variable object X into edible and inedible items, will end up memorizing familiar objects as an association {food(X)} or {toy(X)}.

**Fig. 13:**
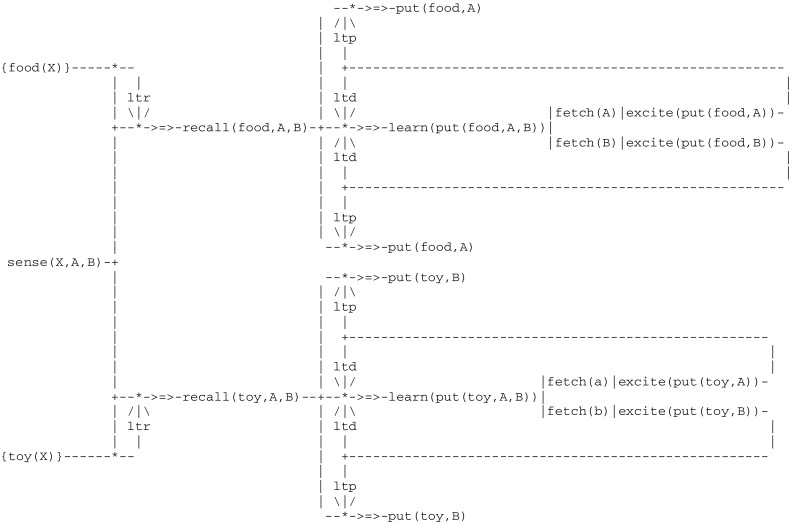
Circuit for sorting familiar object

The second phase (Fig. 13), which leads to sorting objects into one of two bins denoted A and B, starts with a recall from familiar objects. As a result of remembering the category of object X, the sorting process applies to all familiar objects without additional training.

##### Running a simulation

A simulation run for learning the category of objects gives rise to the following log, where inputs from sensors and effector outputs are preceded by a prompt |: and ≫>, respectively:
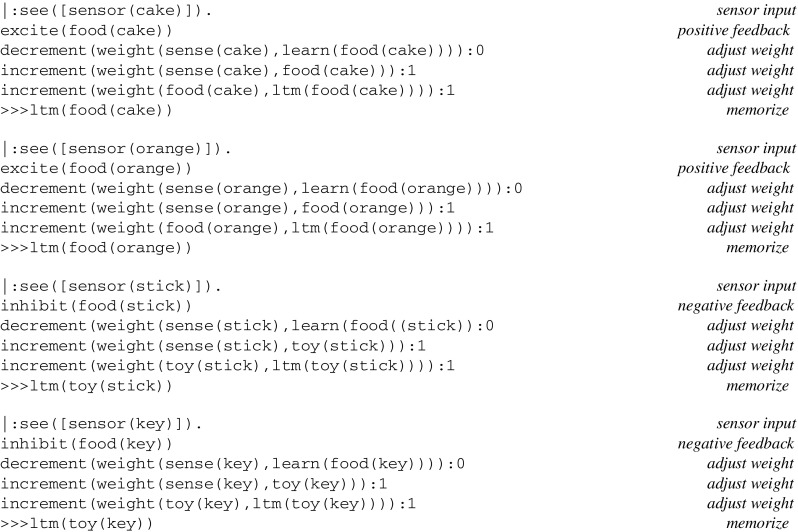
The log of a simulation run for sorting objects is as follows:
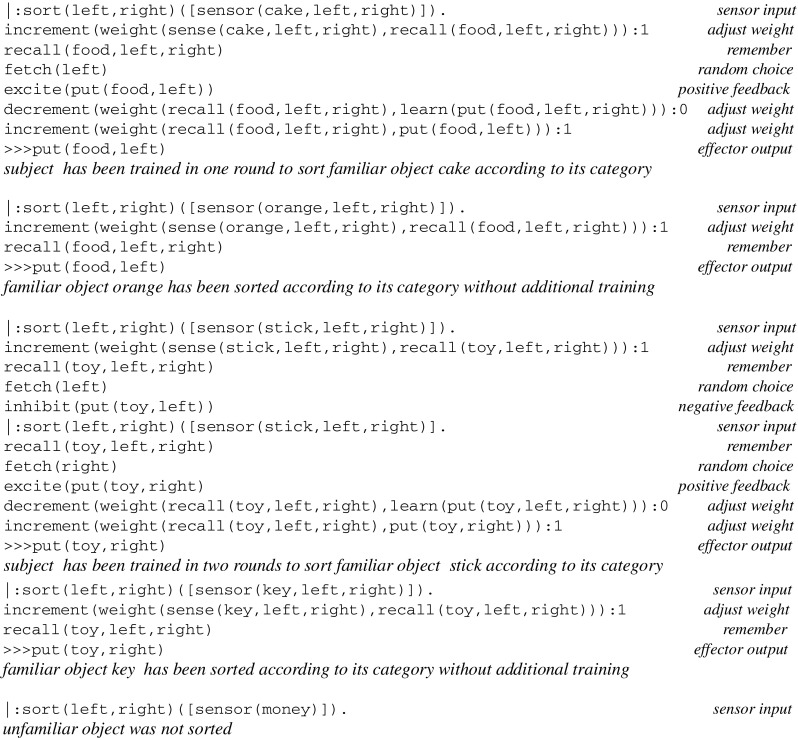


## Discussion

### Comparative approach

A study of some large scale projects (de Garis et al. 2010) reveals a profound disagreement of how to possibly progress towards the goal of reverse engineering a brain in action. While some authors (Markram et al. 2015) reporting about the reconstruction and simulation of a neurological circuitry describe “the emergence of spontaneous spatio-temporal patterns”, some others (Modha et al. 2011) more cautiously believe that *“*the realistic expectation is not that cognitive function will spontaneously emerge” from such simulations, and rather insist that a simulator supplies a substrate within which we can formulate theories of neural computation.

As a first example, the Blue Brain project (Markram 2006) proclaimed objective was “ultimately, to study the steps involved in the emergence of biological intelligence”. Towards this end, they did collect vast amounts of in vitro measurements, and on this basis managed to simulate the current induced by ion channels. By assembling individual neurons, they then reconstructed in silico a neocortical column, i.e., a slice of a rat brain. The interaction of interconnected neurons was then expected to emerge spontaneously, and it did so to a certain extend. This simulated experiment however had no inputs from sensory organs, nor any outputs to other parts of the brain, and as such was not related to any behavior.

SAL, or Synthesis for Leabra and ACT-R (Jilk et al. 2008) was conceived as a merging of two well-established constituents i.e., ACT-R (Anderson et al. 2004), a symbolic production-rule based architecture, and Leabra (O’Reilly and Munakata 2000), a neural modeling system. According to the developers themselves, this integration “is of the simplest form, whereby the visual module in an existing ACT-R model of navigation is replaced with a Leabra vision model, which is capable of processing raw bitmap images in a way that the ACT-R visual module was not capable of doing. Similarly, extant Leabra models are not capable of organizing problem solving behavior”. In ACT-R, operations are purely syntactical without any reference to the semantic content of their representation. Still, it is at this level that learning, memory, and action planning take place. Furthermore, and in accordance with a tradition going back to the theory of the General Problem Solver (Newell and Simon 1976), task representation is also encoded at this level and drives the overall behavior of the model. As a consequence, the resulting integration cannot address the issue of how symbolic representations and/or cognitive functions arise in the brain. This situation is highly illustrative of the inherent shortcomings of present symbolic cognitive models and will be confronted below with our own approach. It is interesting to note here at once that while Jilk et al. (2008) still hope to map the theories either mathematically or in simulated form, they readily add (p. 211) that “the incommensurable categories at the various levels of description will remain necessary to explain the full range of phenomena”.

Somehow at the other end of the wide spectrum of possible integrations, the work of Eliasmith (2013) systematically relates to the semantic content i.e., the information that is contained in groups of spiking neurons. Formally, a set of mathematical methods called NEF (for Neural Engineering Framework) was designed to allow for building spiking neural networks that approximate any nonlinear dynamical system (Eliasmith et al. 2012). The central idea behind the NEF is that a group of spiking neurons can represent a vector space over time and that connections between groups of neurons can compute functions on those vectors. Semantic pointers that somehow correspond to an associative memory do realize a mapping between concept vectors and various known tasks. But as the authors acknowledge themselves, they do not provide a mechanism for how brains learn to represent internal and external states. It is thus unclear how this approach can end up representing cognitive functions. On the positive side however, by providing a normalized interface between this formalism and underlying simulated physiological processes, they have been able to implement the principles of the NEF on both the Neurogrid chip (Choudhary et al. 2012) and the SpiNNaker system (Furber et al. 2014).

### From single neurons to neural assemblies

Since the pioneering work of Hodgkin and Huxley (1952), the usual approach for simulating neural dynamics starts with current flows represented by differential equations. Various proposals have been made to close the gap between the level of individual neurons and higher levels supporting behavior. A possible solution is to consider group of neurons, or *neural assemblies*. Following a tradition going back to D. Hebb (1949) and further illustrated by numerous authors (see e.g., Palm 1982; Edelman 1987; Bienenstock 1994; Knoblauch and Palm 2002; Izhikevich 2006), neural (or Hebbian cell) assemblies can be described informally as groups of strongly interconnected neurons that support specific functions (for a review, see Huyck and Passmore 2013; Pulvermüller et al. 2014). This approach has already led to the design of artifacts relating behaviors and brain processes by mapping neural assemblies onto the topology of brain regions (Seth et al. 2004; Knoblauch et al. 2005). Their existence is generally viewed as resulting from Hebbian learning (Gerstner and Kistler 2002). In their simplest form represented by auto-associative networks, this can lead for example to the creation of local memories (Palm 1980; Knoblauch et al. 2010). In latest models, so-called *operational* cell assemblies allow for the representation of syntactic patterns which are implemented in terms of hetero-associative transition graphs in attractor networks which cause a directed flow of activity through the neural state space (Wennekers and Palm 2009). These assemblies are grounded in, and thus dependent on specific artificial neural network models defining a particular neural state space. This could become critical when confronted with new experimental results (Branco et al. 2010) that have provided a demonstration of the power of dendrites for solving computational problems in the brain. More precisely, it has been found that single dendrites of cortical pyramidal neurons exhibit sensitivity to the sequence of synaptic activation, and thus can encode the temporal sequence of synaptic input. Furthermore, simulation results (Legenstein ad Maass 2011) have confirmed that the branch strength could store a reference to an input pattern, and that a subsequent pattern presentation will elicit reliable spiking of the neuron, resulting in the entire dendritic tree behaving like a network by itself (Costa and Sjöström 2011).

As an alternative, it is proposed here *to model neural assemblies in a simulation framework driven by a virtual machine acting as an interface between neural dynamics and symbolic information defining behaviors*. As a consequence, there will be no reference to any specific neural network model. Whereas in some simulations threads are equated with individual neurons, in others they do represent multiple interconnected neurons whose coordinated activity achieves an aggregated result. Threads thus constitute a general and versatile tool for simulating various levels of structures and/or processes e.g., Hebbian cell assemblies.

### Proposal characteristics

A common way of characterizing cognitive models is given by the two competing paradigms of artificial cognitive architectures (Brooks 1991) i.e., the traditional “*sense*-*think*-*act*” cycle of cognitivist systems, on one side, and the simplified “*sense*-*act*” cycle of *embodied* and/or *emergent* cognition, on the other. Clearly, as explicit in “Top down construction of a virtual machine” section, our proposed model falls into the second category, but it does so by resorting to a kind of symbolic computational framework generally associated with the first approach. This can be related to the hypothesis originally proposed by Newell and Simon (1976) according to which human intelligence can be approximated by a physical symbol system (PSS). According to this hypothesis (see also Nilsson 2007), “*A physical symbol system is a machine that produces through time an evolving collection of symbol structures. (..) An expression designates an object if, given the expression, the system can either affect the object itself or behave in ways dependent on the object*”. Concretely, this means that symbols have to be linked to real objects in two ways i.e., through sensors (the objects providing input to the system) and through effectors (the system acting in return on the objects). Our proposal somehow achieves this. More precisely, it is the concatenation of the pathways leading to the firing of a given thread that allow for the symbols to be connected to the objects. What distinguishes it however from previous implementations (e.g., Newell et al. 1989) is its use of a virtual machine, which constitutes an interface between the physiological and the psychological levels that are associated with both sensing and acting.

Coming back to the analysis of Poggio (2012) alluded to in our Introduction, it is interesting to further confront his views with the models presented in “Examples of mesoscale circuits” section. For instance, he asks “*did intelligence, as the ability to learn, evolve from associative, Pavlov*-*like reflexes and memories, with the addition of (neurally simple) primitive operations such as composition of different memories?*” A detailed look at our models readily reveals that their mesoscale circuits do actually operate just along the lines imagined by this author, with iterated applications of an associative long term memory (ltm) based on long term storage and retrieval (lts/ltr), as introduced in “Associative long term memory (ltm) based on long term storage and retrieval (lts/ltr)” section, playing a key role. This whole approach relies on the direct mapping of perceived *invariant* structures. This mechanism reflects in particular the prime importance of vision as a means of first carving the brain to reflect the reality of the world, and then act on it in return (Barret 2008).

### Potential benefits

Generative models bridging the gap between the physiological and cognitive levels could at the end lead to the discovery of the learning processes involved in the development of cognition. As illustrated in our development of models of animal awareness, our formalism offers a principled guidance towards this goal. More precisely, this is achieved through a two steps process consisting in

- first inducing plausible mesoscale circuits that represent the application of rules such as *matching/oddity to sample*, *win/stay loose/shift*, *recall/sort*, corresponding to the solution of elementary cognitive tasks such as of association, cross-modal integration, etc.
- embedding then these circuits in order to solve higher level tasks such meta-cognition.

The successful application of this methodology could lead to a reconsideration of the whole concept of a “neural code” allowing for relating perception and behavior. Such a neural code may well reside in the spatial arrangement of mesoscale circuit patterns (i.e., a kind of population or sparse coding, as opposed to the more traditional rate or temporal coding associated with spike trains). One might then even consider that there is actually no code at all (in the sense of a specific arrangement always associating the same response to a given stimulus), and that “the code is the overall structure itself”. More precisely, perception might be related to behaviors through the paths found by evolution via iterated hebbian learning.

Another potential benefit of this formalism resides in the insight it offers in support of the recent suggestion that “the operations of the brain are massively asynchronous with respect to each other”. (Zeki 2015). More precisely, as introduced in “Top down construction of a virtual machine” section and formalized in “Computational architecture formal specifications” section, the basic idea here is that there is no central clock in the brain that synchronizes parallel processing systems (i.e., they do have their own local clock), with the activity in each of these systems being reset independently, thus “making of the brain a massively asynchronous organ”.

These assumptions bear strong analogies with the SpiNNaker project (Furber et al. 2014), whose massively parallel computer architecture is inspired by the connectivity of the brain. Indeed, similarly to the tree structure of threads that can be interpreted sequentially and deterministically, the SpiNNaker system can impose deterministic operations in order to match a conventional sequential model under certain condition. More specifically, whereas threads maintain parallel asynchronous communications whose incoming signals are processed individually, the SpiNNaker architecture allows for the transmission of a large number of small data packets obeying a communication protocol according to which

- neurons communicate through action potentials, or ‘‘spikes’’ i.e., asynchronous impulses whose height and width are largely invariant; consequently, information is conveyed only in the *identity* of the neuron that spiked and the *time* at which it spiked
- the information flow in a network can be represented as a time series of neural identifiers; this allows for the encoding of neural activity through the so-called address event representation (AER) information protocol (Boahen 2000).

As a result of these common assumptions, virtual machines interpreting threads could function as an interface allowing for spatio-temporal sequences of spiking neurons to be related to behaviors. In other words, this means that this new simulation tool could be used to simulate both the interface between cell assemblies and the neural level, on one hand, and that between cell assemblies and cognition, on the other.

### Open perspectives

Although our simulation framework must be clearly distinguished from a real brain, it readily offers a macroscopic picture of how brain processes may lead to cognition. Among the many theories we could confront this framework with, we shall concentrate on Edelman’s theory of neural Darwinism (Edelman 1987). The main concept underlying its developments is the so-called *group selection of population.* A first selection process occurring epigenetically during prenatal development leads to a primary repertoire representing the diversity of anatomical connectivity. A subsequent selective process coupled with the subject’s activity results in a second repertoire based on modifications in the strength of synaptic connections reflecting their correlation with signals arising from behavior. Finally, *reentrant* processes “based on the existence of reciprocally neural maps” help to “maintain spatiotemporal continuity in response to real-world interactions”. Although statistical aspects associated with the idea of reentrant processes have led to the development of various artifacts or robots, this highly abstract concept has proved to be difficult to map into more traditional ideas and experimental results. The neurobiological phenomena accounting for them have thus never been observed. It is interesting to note that, in the mind of the author, their existence “obviates the need for explicit exchange of time and place markers of the kind required in parallel computing systems”. In other words, they appear to be a substitution for, or play the role of, the explicit concurrent communicative processes we did strive for in the present article. While the modification of synaptic efficiency associated with the creation of secondary repertoires presumably relies on ltp/ltd processes, we put forward the hypothesis that our proposed complementary lts/ltr processes play a similar role for reentry. In support of this thesis, let us simply confront Edelman (1987) own words: “One of the fundamental tasks of the nervous system is to carry on adaptive perceptual categorization (..). A necessary condition for such perceptual categorization is assumed to be reentry” with the very fact that lts/ltr associative processes were introduced in “Results” in order to implement the concept of a category.

To touch on another, more focused domain of research i.e., that of the origin and nature of consciousness, let us quote Dehaene and Naccache (2001) assessment of the fundamental issues at stake there: “*A complete theory of consciousness should explain (..) what is the range of possible conscious contents, how they map into specific neural circuits, and whether a generic neural mechanism underlies all of them.”* Although our work does not specifically address these questions, our implementation of the third level of animal awareness could still provide some hints about the corresponding sequence of operations:

- potential conscious contents P might have first to be memorized (or directly produced) in {P} via an lts or some other equivalent process.
- a triggering event Q might then be required in order to elicit the retrieval of {P}.
- the association of {P} and Q could finally be made “conscious” in R via an ltr or another equivalent, possibly amplifying process.

The origin of consciousness could thus be found at the level of processing that is shared with “representations of the immediate external environment” (Morsella et al. 2015). Furthermore, in accordance with empirical evidences describing conscious information as being available in a “global workspace” (Baars 2005; Dehaene and Naccache 2001), our protocols associated with higher levels of animal awareness require the broadcast of paths. This could lead to a modeling of this global workspace through a serial *stream of consciousness* (James 1890), whose synchronization with the parallel streams relating perception and behaviors could follow from the introduction of a global clock.

## Conclusion

In summary, as suggested in the introduction, the analytical methods that are used today in computational neuroscience could be complemented with discrete processes aggregating lower level continuous processes in order to relate perception and behavior. Whereas it seems reasonable to consider that at the lower levels there may be valid physical theories, the interaction between higher levels could be described using computer science and/or information systems methods and thus benefit from the results obtained in these domains.

With regard now to a possible “what next?” question, it would be interesting to find out which new constructs, if any, should be added to the present formalism in order to go beyond perceptual categorization e.g., to implement the fourth and fifth levels of animal awareness depicted in (Pepperberg and Lynn 2000). If indeed, as speculated in (Carew 2002; Poggio 2012) and supported by the models presented above, “classical and operand conditioning have in common, an exciting principle might emerge: evolution may have come up with a neural ‘associative cassette’ that can be used in either type of conditioning, depending of the neural circuit in which it is embedded”. In other terms, the lts/ltr pair might be a candidate for the role of the canonical microcircuit looked for in (Modha et al. 2011). Finally yet, if ltp/ltd threads have been explained at the light of the so-called *spike*-*time dependent plasticity,* or STDP (Markram et al. 1997; Brette et al. 2007), their extension into hypothetical lts/ltr threads raises the issue of their possible grounding into actual biological processes.
